# Measuring job stress in transportation workers: psychometric properties, convergent validity and reliability of the ERI and JCQ among professional drivers

**DOI:** 10.1186/s12889-021-11575-1

**Published:** 2021-08-28

**Authors:** Sergio A. Useche, Francisco Alonso, Boris Cendales, Luis Montoro, Javier Llamazares

**Affiliations:** 1 Faculty of Psychology, University of Valencia, Valencia, Spain; 2 DATS (Development and Advising in Traffic Safety) Research Group, INTRAS (Research Institute on Traffic and Road Safety), University of Valencia, Valencia, Spain; 3 Faculty of Economic and Administrative Sciences, El Bosque University, Bogotá, Colombia; 4 FACTHUM. Lab (Human Factor and Road Safety) Research Group, INTRAS (Research Institute on Traffic and Road Safety), University of Valencia, Valencia, Spain; 5 Department of Technology, ESIC Business and Marketing School, Madrid, Spain

**Keywords:** Job stress, Job demand-control-model, Effort-reward imbalance, JCQ, ERI, Transportation workers, Professional drivers

## Abstract

**Background:**

The accumulated evidence has shown how professional drivers are, in psychosocial terms, among the most vulnerable workforces, and how their crashes (some of them preceded by stressful working conditions) constitute both an occupational and public health concern. However, there is a clear lack of validated tools for measuring stress and other key hazardous issues affecting transport workers, and most of the existing ones, frequently generic, do not fully consider the specific features that properly describe the work environment of professional driving. This study assessed the psychometric properties, convergent validity and consistency of two measures used for researching occupational stress among professional drivers: the Siegrist’s ERI (Effort-Reward Imbalance Inventory) and Karasek’s JCQ (Job Content Questionnaire).

**Methods:**

We examined the data collected from 726 Spanish professional drivers. Analyses were performed using Structural Equation Models, thus obtaining basic psychometric properties of both measures and an optimized structure for the instruments, in addition to testing their convergent validity.

**Results:**

The results suggest that the abbreviated versions of ERI (10 items) and JCQ (20 items) have clear dimensional structures, high factorial weights, internal consistency and an improved fit to the task’s dynamics and hazards, commonly faced by of professional drivers; a short set of items with low psychometrical adjustment was excluded, and the root structure of the questionnaires was kept.

**Conclusions:**

This study supports the value and reliability of ERI-10 and JCQ-20 for measuring job stress among professional drivers. Also, there is a high consistency between both measures of stress, even though they belong to different theoretical conceptions of the phenomenon. In practical settings, these instruments can be useful for occupational researchers and practitioners studying stress-related issues from the perspective of human factors.

## Key points

- This study details the validation of two relevant questionnaires for assessing job stress in professional drivers: Effort-Reward Imbalance (ERI) inventory and Job Content Questionnaire (JCQ).

- These self-reported questionnaires present adequate dimensional structures, factorial weights and internal consistencies among professional drivers.

- The core dimensions and indexes provided by ERI and JCQ have an adequate convergent validity with criterion variables on psychosocial factors at work, health and occupational safety.

- The scales validated can be useful for occupational health research, and for the designing of evidence-based interventions in the industry of transportation.

## Background

Overall, worldwide evidence supports the fact that job stress represents a key psychosocial risk factor in most occupations [1, 2]. Job stress constitutes an issue whose implications involve not only the workers’ performance, but their health, safety, and well-being [2–4]. In the particular case of transportation workers, the interest in studying job stress-related dynamics has been gaining ground during the last decade, due to the fact that this type of stress affects not only the workers’ domain but, given its predictive role of traffic crashes, threatens the health and welfare of all potential users of the roads [4, 5].

Accordingly, different authors consider occupational risks of professional drivers a public health problem [3, 6, 7]. This is due to the fact that, beyond the individual effect of adverse working conditions, work-related health and behavioral outcomes (e.g., psychological strain, sleep disorders and risky behaviors) may compromise safe driving outcomes. Globally, up to a third of all traffic accidents, which cause 1.3 million deaths a year, involve professional drivers [8, 9]. Therefore, developing reliable approaches for addressing job stress is an essential first step for designing occupational health and safety-related interventions aimed at reducing the great burden of occupational risk factors in the transportation industry, and their negative impact on community health.

Among all work stress-related measurement approaches, the Effort-Reward Imbalance (ERI) [10] and the Job Demand-Control (JDC) [11] models constitute two relevant perspectives, whose assumptions have been previously tested in several industries, endorsing their reliability, consistency and usefulness for research in occupational health [12–15].

On one hand, the ERI model [2, 16] supports the idea that workers perceive a set of extrinsic and intrinsic rewards as a result of the efforts invested in their job. However, an imbalance between the efforts made and the obtained rewards may lead them to a state of “active distress” [17]. On the other hand, the Job Demand-Control model (JDC) [11] defines job strain as a condition in which quantitatively elevated and conflicting demands are combined with scarce decision authority and skill discretion (*control*). Among transportation workers, typical job features such as repetitive and monotonous work, ergonomic demands, inflexible schedules, shift work and excessive environmental stimuli may enhance the observed high prevalence of job strain, as documented by various recent studies [5, 18, 19].

In this regard, similar adverse health, performance and safety-related outcomes have been documented for the case of both ERI and JDC models [2, 17, 20], such as cardiovascular diseases [2, 21], acute and chronic fatigue [6], poor sleep quality [22, 23], anxiety and depression [24–26]. Moreover, other psychosocial hazardous outcomes at work, such as burnout [27], job dissatisfaction [28] and absenteeism [29] have shown close relationships with workplace stress, implying considerably elevated human and economic costs for organizations and healthcare systems [30].

### The current study

Bearing in mind the aforementioned considerations, and the growing need for validated instruments that address work-related hazards in highly vulnerable workforces, the main purpose of this paper was to assess the psychometric properties, convergent validity and internal consistency of two measures for occupational stress research among professional drivers: the Siegrist’s ERI (Effort-Reward Imbalance Inventory) [10] and Karasek’s JCQ (Job Content Questionnaire) [11].

Given that many previous experiences applying these questionnaires in different workforces support their reliability, consistency and validity, in addition to their adaptability to different work environments, it was hypothesized that both ERI and JCQ will present a good fit to the data and adequate factor loadings. Also, it was expected that they will keep their generic factor structure, even though some minor variations in the item composition might take place, if we consider the set of task-related particularities of professional driving, such as an expected low variability in skill discretion and autonomy within their work environment.

## Methods

### Sample

This research used a convenience sample of Spanish professional drivers from all 17 regions of the country. The sample size was estimated through an a priori lower bound sample size calculation for structural equation models [31, 32]. The minimum sample size for a model with an anticipated effect size of .1 (considered a low effect size), a statistical power level of .8, three latent variables (demands, decision latitude and social support factors of the JCQ), 20 observed variables (JCQ items) and a Probability level of 0.05, was *n* = 323 participants. An attempt was made to at least double this number in order to ensure adequate statistical power for the study (rather than for the sample representativeness), increasing the number of participants up to the final size of *n* = 726 individuals, after listwise discarding 28 (< 4%) cases due to partial completion of the questionnaire. Also, and although the number of items/variables is not an adequate core criterion to establish minimum sample sizes, the proportionality between the sample size and the questionnaire length was considered, being approximately 7:1 (or seven subjects per questionnaire item), which is higher than the minimum 5:1 usually recommended for EFA/CFA-SEM procedures [33, 34]. However, the survey was not excessively long; it had a total of 106 items, so that respondents did not get tired nor lost motivation during its completion, as suggested in key health research guidelines [35, 36].

The full sample was composed of professional drivers aged between 24 (minimum) and 70 (maximum), with a mean age of *M* = 47.1 (*SD* = 8.05) years. Regarding gender, and as it was predictable, we found a huge overrepresentation of male workers in this industry: 98.6% of them were men and 1.4% women.

The mean hourly intensity of driving during a week timetable was *M =* 7.82 (*SD* = 1.92) hours/day and the average number of weekdays working (driving) was *M =* 5.23 (*SD* = .69) days. As for road crash records, the average number of occupational traffic crashes suffered during the last 2 years, regardless of their severity, was *M =* .40 (*SD* = 1.04). Further key demographic and job-related data of the participants of the study are presented in detail in Table 1.

**Table 1:** Demographic and driving work-related information of the study sample

Feature	Category	Frequency	Percentage
Gender	Female	10	1.4%
	Male	713	98.6%
Days working (driving) a week	3 or less	8	1.1%
	4	21	2.9%
	5	493	67.9%
	6	162	22.3%
	7	21	2.9%
	No regularity	21	2.9%
Hours driving per day	< 5 h	49	6.7%
	5–8 h	387	53.3%
	9–12 h	261	35.9%
	> 12 h	6	0.9%
	No regularity	23	3.2%
Shift working	Yes	324	44.6%
	No	382	52.6%
	Only exceptionally	20	2.8%
Transportation modality	Passenger	168	23.1%
	Cargo	521	71.8%
	Other	37	5.1%
Type of vehicle	Urban Bus	31	4.3%
	Intercity Bus	121	16.7%
	Van or smaller company vehicle	57	7.8%
	Long-haul / freight vehicle	486	66.9%
	Other	31	4.3%

### Study design and procedure

This was a transversal (or cross-sectional) research. In order to carry out this study, framed within a larger collaborative research project in cooperation with organizations in the field of transportation and with Spanish associations of professional drivers, potential participants were allocated and invited to partake through their organizations or associations. This means that a non-probabilistic (convenience) sampling method was employed, as in other similar studies focused on specific workforces. As for the data collection procedure setting, all partaking drivers were asked to complete the questionnaire throughout a period of approximately 1 hour during their formation courses, as previously agreed by their respective companies; this enhanced the disposition of an adequate physical environment for the task. Also, a member of the research staff was permanently monitoring the completion of the questionnaires, in order to solve potential doubts or answering questions of participants, who were previously informed about the protection of their personal data by means of an informed consent form (see *Ethics*). Special emphasis was put on the fact that the data would be only used for research and scientific purposes. The overall number of incomplete/illegible (excluded) questionnaires were < 30 and the response rate was around 80% and, which means four out of five drivers that were invited accepted to participate and filled out the research questionnaire.

### Description of the questionnaire

For this study, we used a self-report questionnaire that was forward-translated from English to Spanish and backward-translated from Spanish to English by two independent professional translators, in order to ensure the accuracy of the translation, as it is often suggested in literature [35]. Afterwards, both versions of the study questionnaire were reviewed by two experts in the research topic (*Expert 1:* on job stress measuring, and *Expert 2:* on psychometrics in occupational health), who approved the final form of the survey to be delivered to participants, as advised by the STROBE (*STrengthening the Reporting of OBservational studies in Epidemiology*) and COSMIN (*COnsensus-based Standards for the selection of health status Measurement INstruments*) checklists, created for this purpose [35–38].

The full version of the questionnaire was composed of three core sections:

The first section comprised *a)* demographic variables, e.g., gender, age, town/city of residence, type of job, and *b)* work-related features, e.g., type of vehicle(s) driven at job, transport modalities (cargo, passenger or other), hours driven per day, days working per week, shift-working or stability of work shifts, and occupational driving safety indicators, i.e., traffic crashes suffered along the last 2 years during occupational shifts. It is important to remark that, in the Spanish legislation, accidents suffered during in-itinere displacements (from the place of residence to work and vice versa) are also considered occupational accidents, so this rate includes both in-itinere and on-duty traffic crashes and it was used as a criterion variable (see Table 6 for more information).

The second part of the questionnaire presented the two questionnaires to validate: firstly, we used the Effort-Reward Imbalance Inventory (ERI) [10] in its short/10-item version, that was previously translated into Spanish, which has already been adapted and used in several applied studies dealing with workers with different occupations, including samples of professional drivers from different countries [39, 40]. The questionnaire is composed of two core sub-scales, used to assess psychosocial, stress-related risk factors at work according to the factors proposed in the Effort-Reward Imbalance model [41]. The model points out the imbalance between two sub-scales as an indicator of job stress: extrinsic effort (commonly labeled as *Efforts*; 3 items, α = .74 original) and perceived rewards (commonly labeled as *Rewards*; 7 items, α = .79 original). Further details on scoring of this version of the ERI are presented in Table 3. Throughout two previous empirical studies, the ERI has shown consistent results and good predictive power for adverse psychosocial and health outcomes among workers, such as an impaired overall mental health status, burnout and musculoskeletal symptoms [13, 42]. Then, we used the 22-item version of the Job Content Questionnaire (JCQ) [11] in its Spanish version, previously validated by Gómez (2011) among Hispanic workers [43]. This version of the scale, used to assess psychosocial factors at work that could potentially lead to job strain, conceived as the job stress indicator of the Job Demand-Control model, comprises the following subscales: skill discretion (6 items, α = .50 original) and decision authority (3 items, α = .61 original), whose sum allows for the calculation of the variables “control” (α = .65 original); psychological demands (5 items, α = .67 original); supervisor/manager support (4 items, α = .78 original); and peer/co-worker support (4 items, α = .72 original). Additionally, general social support (α = .83) can be calculated as the sum of peer and supervisor support. Further details on the scoring of this version of the ERI are presented in Table 5. Job strain has been empirically associated with health outcomes of workers belonging to different industries [20, 29] and, more specifically, its predictive value for both health problems [19, 21] and safety records [44–46] has also been assessed among professional drivers.

As for the third part of the questionnaire, and apart from the occupational driving-crash rate, two supplementary questionnaires were chosen as criterion variables, in order to test the convergent validity of ERI and JCQ:

*a)* The abbreviated version of the Copenhagen Psychosocial Questionnaire III (COPSOQ-III). The COPSOQ series of questionnaires were initially developed by Kristensen, Hannerz, Høgh & Borg [26] and updated by Nübling et al. [47]. The COPSOQ tool is widely used for workplace psychosocial risk assessment and organizational development. It constitutes a generic instrument, which can be potentially used for all types of jobs, in any industry and for workplaces of different sizes [47]. The third version of the questionnaire was lately validated for Spanish professional drivers by Useche, Montoro, Alonso & Pastor [46]. For this version, the self-report inventory is composed of 52 items measured on a scale from 1 = “never/hardly ever” or “to a very small extent”, to 5 = “always”, or “to a very large extent”, that are distributed along various factors or sub-scales:

Demands (F1), composed of 12 items (α = .92; example item: *“Do you have to work very fast?”*); Influence and development (F2), consisting of 6 items (α = .85; example item: “*Do you have the possibility of learning 7 new things through your work?”*); Interpersonal relations and leadership (F3), containing 13 items (α = .91; example item: *“Is there good co-operation between your colleagues at work?”*); Job insecurity (F4) composed of 6 items (α = .85; example item: *“Are you worried about new technology making you/your work redundant?”*); and Strain - effects and outcomes (F5) consisting of 15 items (α = .90; example item*: “How often have you thought about giving up your profession?”*). Each one of these factors provides a continuous score, obtained through adding the punctuation of their items, and can be treated either as continuous variables or qualitatively analyzed, if the sample is small, or if a case study needs to be performed.

Also, the COPSOQ-III includes an additional item for workers to report their self-rated health status in a raw scale 0 (very bad health status) to 10 (very good health status) [47], that was also incorporated as a criterion variable.

*b)* The short version of the General Health Questionnaire (GHQ-12) [48], a 12-item Likert questionnaire aimed at assessing different symptoms that might potentially affect the mental health of individuals, using four different levels to assess the frequency of each symptom of discomfort (1 = never/rarely; 4 = very often/always). This scale can be scored in a single factor widely known as *psychological distress* (α = .74), with the possible values ranging between 12 (very low degree of psychological distress) and 48 (very high psychological distress).

### Data processing (statistical analysis)

Initially, a data curation was performed. As only a very reduced number (< 4%) of the received forms were incomplete or illegible, only fully filled questionnaires were considered for this study, using listwise deletion for filtering the missing data; although one of the shortcomings of listwise deletion is that it may substantially shorten the sample size (and statistical power could be lost [49]), in this case (i) the ratio of questionnaires with missing data was minimal, and (ii) the sample remained considerably large, as it is mentioned in the sample subsection. Afterwards, a basic data and coding was carried out, allowing us to perform descriptive analytic procedures. The factorial structures of the ERI and JCQ were respectively assessed through Exploratory and Confirmatory Factor Analyses (EFA and CFA, respectively) and sequentially tested. The exploratory analyses used a maximum likelihood (ML) method with Promax oblique rotations (please see Table 7 in Appendix 1 ). As for the CFA, and based on the available theoretical and empirical support on the validated instruments, this study is based on confirmatory models, that entail several advantages as for the management of missing data, categorical and non-normally distributed variables [50]. For descriptive analyses and exploratory analyses, the IBM SPSS software (version 26) was used, while *lavaan* “latent variable analysis” R-based software (version 0.6–5) was used for specifying and estimating the models. Weighted Least Square Mean and Variance adjusted (WLSMV) estimations were applied, keeping in mind that the data was predominantly ordinal and did not meet multivariate normality.

As suggested by expert studies, the model fit was weighed by means of several (instead of single) estimators [51]: Chi-square (*χ*^*2*^), Confirmatory Fit Index (CFI), Normed Fit Index (NFI) and Root Mean Square Error of Approximation (RMSEA). The model fit was founded on the cut-off standards most commonly used in literature: a CFI/NFI higher than .90 and a RMSEA lower than .08 suggest a reasonable model fit. Also, the convergent validity of both questionnaires was tested by means of three selected Criterion Variables (CVs) supported in the literature (see *Description of the Questionnaire* for further information). For this purpose, Spearman’s *rho* (or *r*_*s*_) bivariate correlations, performed using the full sample, were used to assess the association measures among pairs of study variables, considering their robustness over Pearson’s (*r*) correlations when ordinal values are analyzed [52].

Finally, the reliability (or internal consistency) of the scale and its items was gauged through *1)* Cronbach’s alpha coefficients *(α),* and *2)* the Composite Reliability Index (CRI), an additional consistency index that ranges between 0 (no consistency) and 1 (total consistency), statistically founded on the factor loadings and residuals observed in the confirmatory results. The use of this second index also helps to overcome some of the traditional gaps of Cronbach’s alpha as a single way for assessing scale reliability [53].

## Results

### Structural models

With the aim of understanding the factorial structure of the Spanish versions of the ERI and JCQ, Factor Analyses were performed. First, we tested the fit of the data to an Exploratory Factor Analyses (EFA) for both instruments (item factorial weights are shown in Table 8 in Appendix 1), finding a reasonable adjustment for both ERI (2 factors; all item *λs >* 0.30; 54.011% of variance explained) and JCQ (5 factors; 62.03% of variance explained).

These rotated solutions are interpretable and theoretically sensitive [54]. In particular, the JCQ’s five-factor solution results consistent with previous validation studies [55, 56], in which there is a general consensus on its factorial structure [55, 57, 58]. Likewise, the ERI bifactorial solution is consistent with the general trend in previous European validation studies [59, 60], which for abbreviated versions of the questionnaire coincide in the identification of at least two fixed factors (i.e., effort and rewards). Furthermore, using the EFA scree plots as criteria for factor extraction (see Figs. 3 and 4 in Appendix 1 for plots and exploratory factor loadings), it was found that, in the case of the JCQ, the models with one to five factors produce eigenvalues greater than 1; and in the case of the ERI, the bifactorial model is the only one that produces eigenvalues greater than 1. Taking these theoretical and statistical criteria into account, in this study the JCQ 5-factor solution and ERI 2-factor solution were chosen to test them using CFA.

#### Effort-reward imbalance inventory (ERI)

**Fig. 1:**
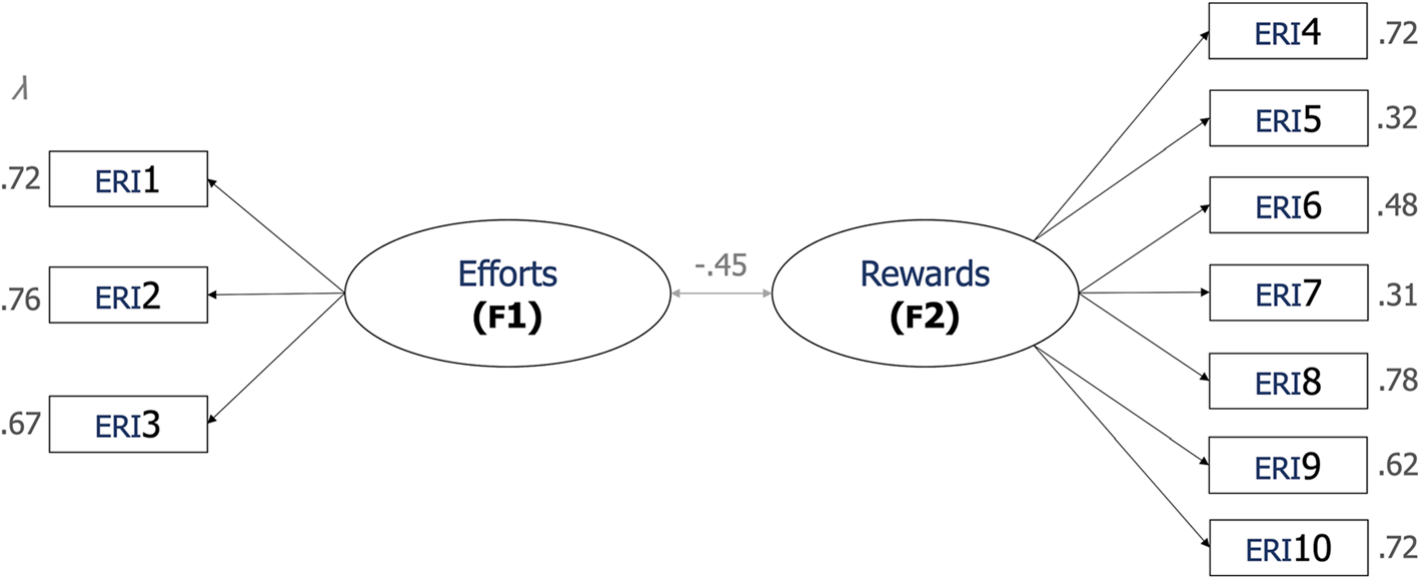
Standardized parameter estimates. Notes: All estimates were *p <* 0.001; the numbers within squares represent the original numbers of the items in the ERI (as shown in Table 2)

The original structure of the short version of the ERI is composed of two factors: Efforts (F1) and Rewards (F2). Thus, the baseline two-factor model was tested, showing relatively good but improvable fit indexes, with: *χ*^2^(34) = 360.049, *p <* .001, CMIN/DF = 10.590; RMSEA = .115 with 90% CI of .104–.126; CFI = .849; and NFI = .837. A close inspection of this baseline two-factor model allowed us to identify a reduced set of very large modification indexes that pointed out a relevant relationship between some items, used for constraining the model. The new simplified model fitted the data reasonably well, presenting the following fit indices: *χ*^2^(25) = 117.780, *p <* .001, CMIN/DF = 4.711; RMSEA = .072 with 90% CI of .059–.085; CFI = .957; and NFI = .947. Compared to the baseline model, the final two-factor structure presents a much better fit without the need of deleting questions, bearing in mind both the considerably adequate factor loadings (all *λ >* 0.30) and the reliability scores obtained in the following analysis (see *3.2 Internal consistencies*). Table 2 shows the content, descriptive data (average scores and standard deviations), standardized factor loadings and significance levels of each one of the items composing the ERI-10, as it is also presented in Fig. 1.

**Table 2:** ERI-10 structure. Item content, factor the item belongs to, standardized factor loading (**λ**), standard error (S.E.), critical ratios (C.R.) and *p*-values in the retained model

Item	Item Content	Factor	M^a^	S.D.^b^	λ	S.E.	C.R.	P
ERI1	I have constant time pressure due to a heavy workload.	Efforts (F1)	3.03	.82	.724	0.076	13.766	<.001
ERI2	I have many interruptions and disturbances while performing my job.		2.54	.80	.757	0.069	14.777	<.001
ERI3	Over the past few years, my job has become more and more demanding.		2.71	.86	.668	0.067	14.418	<.001
ERI4	I receive the respect I deserve from my superior or a respective relevant person.	Rewards (F2)	2.34	.88	.725	0.061	17.042	<.001
ERI5^(−)^	My job promotion prospects are poor.		2.05	.92	.321	0.059	7.849	<.001
ERI6^(−)^	I have experienced or I expect to experience an undesirable change in my work situation.		2.36	.94	.480	0.061	11.620	<.001
ERI7^(−)^	My job security is poor.		2.57	.91	.313	0.058	7.650	<.001
ERI8	Considering all my efforts and achievements, I receive the respect and prestige I deserve at work.		2.27	.87	.782	0.059	18.083	<.001
ERI9	Considering all my efforts and achievements, my job promotion prospects are adequate.		2.28	.88	.619	0.058	14.843	<.001
ERI10	Considering all my efforts and achievements, my salary/income is adequate.		2.04	.85	.722	0.056	17.029	<.001

*Notes:*^(−)^ Negative Item; ^a^ Arithmetic mean; ^b^ Standard deviation

#### Scoring and calculation of effort-reward imbalance ratio

This version of the ERI keeps the item number and basic structure of the previous version, containing 3 items within Efforts – F1, and 7 items within Rewards – F2, that should be computed for calculating the imbalance between Efforts and Rewards. These two root factors of this version of the ERI are calculated as the sum of their respective group of items, once the negative questions had been decoded, as shown in the fourth column of Table 3.

**Table 3:** ERI-10 factor scoring parameters and variable ranges

Variable/Factor	No.	Number of items	Items involved	Calculation formula	Range
*Root Factors*					
Efforts	F1	3	1,2,3	(ERI1 + ERI2 + ERI3)	[3–12]
Rewards	F2	7	4,5,6,7,8,9,10	(ERI4 + (5-ERI5) + (5-ERI6) + (5-ERI7) + ERI8 + ERI9 + ERI10)^a^	[7–28]
*Effort-Reward Imbalance (ERI) Indicator*^*b*^					
Effort-Reward Imbalance (ERI)	F3	10	F1,F2	K*(F1/F2) = ((7/3)*(F1/F2))^c^	–

*Notes:*^a^Assuming that items ERI5, ERI6 and ERI7 (measured in the scale 1–4) have not been previously reversed; ^b^Represents the imbalance ratio between Efforts and Rewards, or *ERI*; ^c^Being *K* = (# items on F2/ # items on F1)

#### Job content questionnaire (JCQ)

The original structure of this version of the JCQ is composed of 22 items, distributed in five item-based factors: Skill Discretion (F1), Decision Authority (F2), Psychological Job Demands (F3), Supervisor Support (F4), and Co-worker/Peer Support (F5). Firstly, the baseline five-factor model was tested using Confirmatory Factor Analysis (CFA), showing considerably adequate, well-fitted indexes, with: *χ*^2^(199) = 1046.902, *p <* .001, CMIN/DF = 5.261; RMSEA = .077 with 90% CI of .072–.081; CFI = .857; and NFI = .831. Nevertheless, a close inspection of this baseline five-factor model allowed us to detect a short set of very large modification indexes that pointed out a relevant relationship between some items, used for constraining the model. Also, and bearing in mind that the original instrument has 22 items for assessing the aforementioned five main factors, we decided to clear the scale by excluding those items which reported obvious psychometric issues in the measurement of their respective constructs, including those items with factorial loadings *(****λ****)* under .30.

**Fig. 2:**
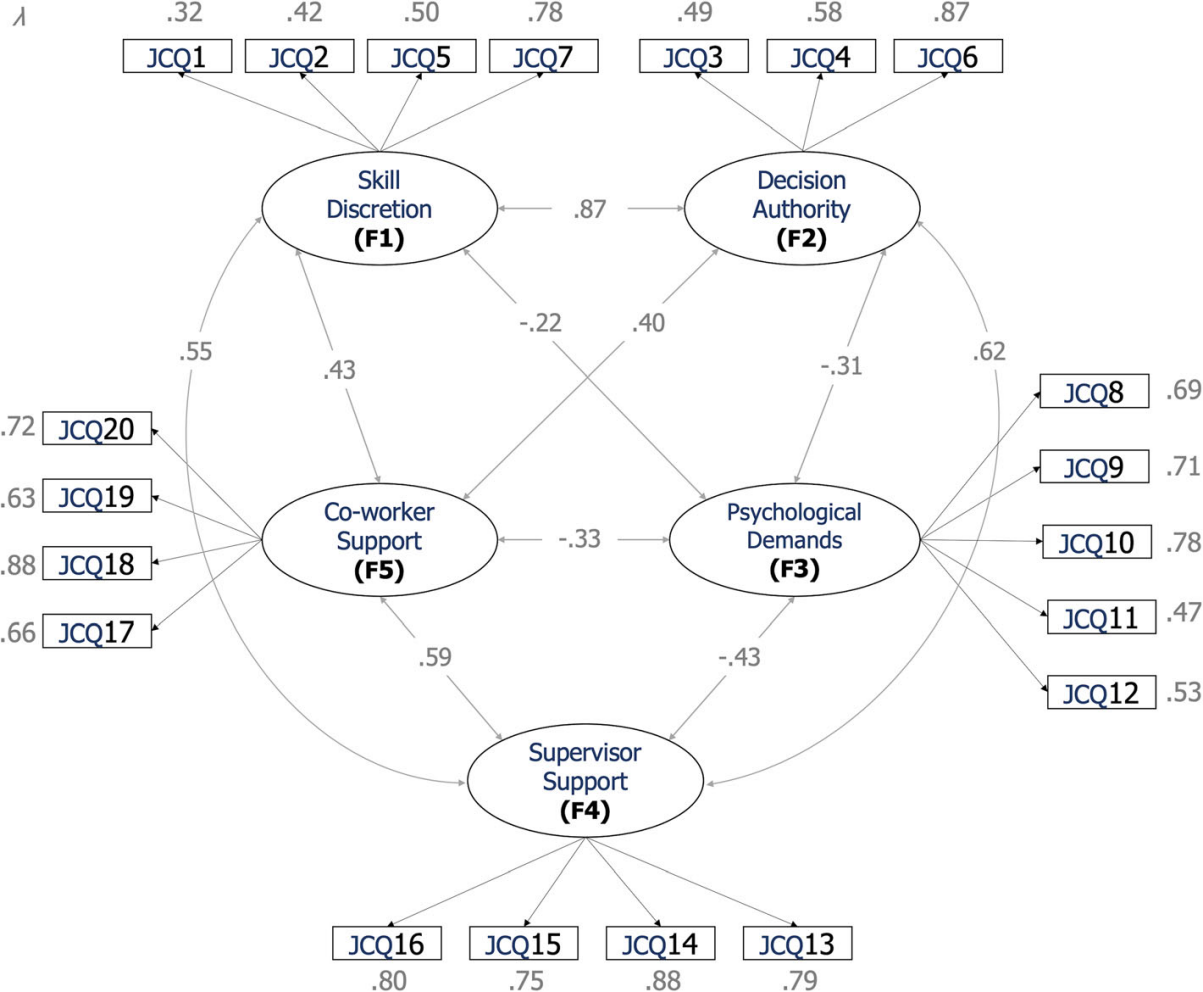
Standardized parameter estimates. Notes: All estimates were *p <* 0.001; the numbers within squares represent the numbers of the items in the shortened version of the JCQ (as shown in Table 4)

**Fig. 3:**
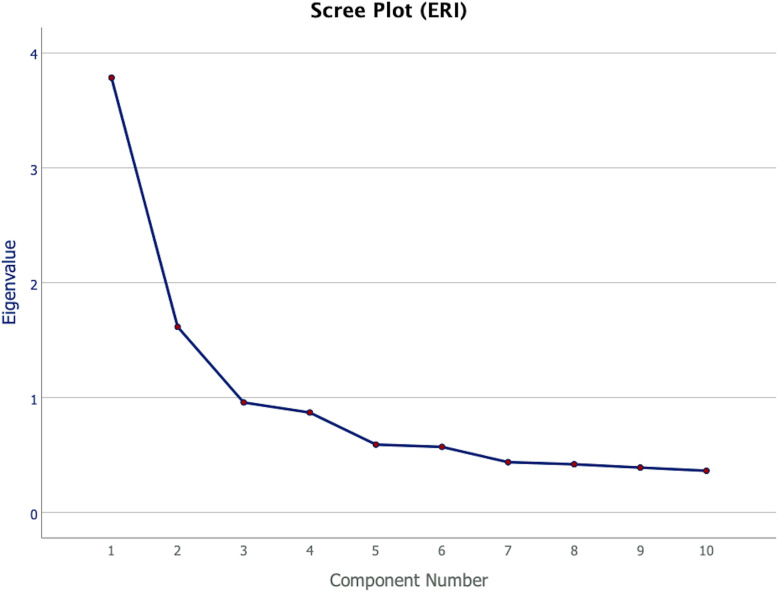
Scree plot (EFA) – Effort-Reward Imbalance inventory

**Fig. 4:**
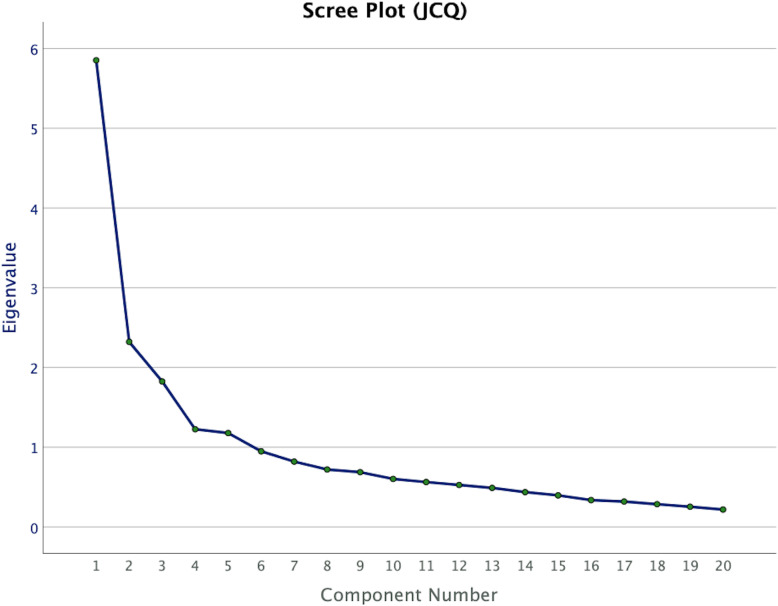
Scree plot (EFA) – Job Content Questionnaire

CFA outcomes can indicate that a model is not acceptable for reasons such as insignificant indicators or items with deficient psychometric adjustment, so it must be modified and improved considering certain factors, such as factor loadings and drawn covariances, and the model’s fit to the data should be tested a second time. Accordingly, two items of the F1 (*Skill Discretion*) were dismissed. The new simplified model fitted the data reasonably well, presenting the following fit indices: *χ*^2^(153) = 477.392, *p <* .001, CMIN/DF = 3.120; RMSEA = .054 with 90% CI of .049–.060; CFI = .943; and NFI = .918. It is relevant to remark that, compared to the baseline model including these two items, the final five-factor structure with 20 items presents a much better fit, considering both the adequate factor loadings of all the remaining items (*λ >* 0.30) and the reliability scores obtained in the next step (see *3.2 Internal consistencies*). Table 4 shows the content, descriptive data (average scores and standard deviations), standardized factor loadings and significance levels of each one of the items composing the JCQ-20, as shown in Fig. 2 as well.

**Table 4:** JCQ-20 structure. Item content, factor that the item belongs to, standardized factor loading (**λ**), standard error (S.E.), critical ratios (C.R.) and *p*-values in the retained model

Item	Item Content	Factor	M^a^	S.D.^b^	λ	S.E.	C.R.	P
JCQ1	I need to be learning new things.	Skill Discretion (F1)	3.28	.70	.32	.05	7.07	<.001
JCQ2	I need to be creative.		2.85	.78	.42	.24	6.19	<.001
JCQ5	There is variety in the activities I do.		2.70	.81	.50	.28	6.53	<.001
JCQ7	I have the opportunity to develop my own skills.		2.78	.82	.78	.40	7.07	<.001
JCQ3	I can make many decisions by myself.	Decision Authority (F2)	3.03	.83	.49	.04	11.97	<.001
JCQ4	I have a lot of freedom to decide how to do my job.		2.67	.87	.58	.06	12.58	<.001
JCQ6	My opinions count a lot.		2.55	.89	.87	.16	11.97	<.001
JCQ8	I have to work very fast.	Psychological Job Demands (F3)	2.88	.86	.69	.10	12.41	<.001
JCQ9	I have to work very hard.		3.01	.82	.71	.06	15.63	<.001
JCQ10	I am asked to do an excessive amount of work.		2.64	.92	.78	.07	16.99	<.001
JCQ11(−)	I have enough time to do my job.		2.40	.78	.47	.06	10.61	<.001
JCQ12	I have to respond to contradictory orders.		2.58	.89	.53	.06	12.41	<.001
JCQ13	My boss or supervisor cares about the economic well-being of the staff in charge.	Supervisor Support (F4)	2.26	.96	.79	.05	21.02	<.001
JCQ14	My boss or supervisor pays attention to what I say.		2.59	.90	.88	.04	24.35	<.001
JCQ15	My supervisor or boss helps to get the job done.		2.76	.84	.75	.04	20.97	<.001
JCQ16	My supervisor is successful in getting you to work well in a team.		2.66	.87	.80	.04	21.02	<.001
JCQ17	The people I work with are competent to do their job.	Co-worker Support (F5)	2.87	.76	.66	.07	13.85	<.001
JCQ18	The people I work with are interested in me personally.		2.50	.87	.88	.09	16.34	<.001
JCQ19	My coworkers are friendly.		2.98	.69	.63	.06	14.50	<.001
JCQ20	My colleagues help to get the job done.		2.87	.77	.72	.08	13.85	<.001

*Notes*: ^(−)^ Negative Item; ^a^ Arithmetic mean; ^b^ Standard deviation

#### Scoring and calculation of the job strain index

This version of the instrument keeps the same structure as the previous version, and the possible scoring ranges in the Likert scale 1–4 guarantee the parity between Psychological Demands and Control for the calculation of the Job Strain (JS) indicator. This suggests an imbalance between the aforementioned two factors if the JS score is higher than 1.0. Both specific scores in Social Support (from supervisors – F4, and from co-workers – F5) and the general Social Support score – F7 can be used for further comparisons, data crossing and further analyses. The raw values of the five root factors of the JCQ-20 are calculated as shown in the fourth column of Table 5.

**Table 5:** JCQ-20 factor scoring parameters and variable ranges

Variable/factor	No.	Number of items	Items /factors involved	Calculation formula	Range
*Root Factors*					
Skill Discretion	F1	4	1,2,5,7	((JCQ1 + JCQ2 + JCQ5 + JCQ7)*3)	[12–48]
Decision Authority	F2	3	3,4,6	((JCQ3 + JCQ4 + JCQ6)*4)	[12–48]
Psychological Demands	F3	5	8,9,10,11,12	((JCQ8 + JCQ9)*3) + ((JCQ10 + JCQ12)*3) + ((5-JCQ11)*2)^a^	[12–48]
Supervisor Support	F4	4	13,14,15,16	(JCQ13 + JCQ14 + JCQ15 + JCQ16)	[4–16]
Co-worker Support	F5	4	17,18,19,20	(JCQ17 + JCQ18 + JCQ19 + JCQ20)	[4–16]
*Composed Factors*^*b*^					
Control	F6	7	F1,F2	F1 + F2	[24–96]
Social Support	F7	8	F4,F5	F4 + F5	[8–32]
*Job Strain (JS) Indicator*^*c*^					
Job Strain	F8	12	F3,F6	(F3*2)/F6	–

*Notes:*^a^Assuming that the negative item (JCQ11, measured in a 1–4 scale) has not been previously reversed; ^b^Can be understood as the sum of previously calculated factors with acceptable psychometric properties; ^c^A resulting value higher than 1.0 indicates imbalance between Demands and Control, or *Job Strain*

### Internal consistencies

Alpha estimates were (except for the JCQ’s *Skill Discretion* scale, with α = .63, that is acceptable although not optimal) all above the usual α = .70 criterion that is a popular rule of thumb advised in many sources as an indicator of adequate internal reliability [61]. Moreover, the Composite Reliability Index (CRI) was also assessed, in order to provide a measure complementary to the Cronbach’s Alpha, showing highly adequate reliabilities for all the three latent constructs addressed by both instruments, as follows:

#### Reliability and consistency of ERI-10

In the case of the Effort-Reward Imbalance Inventory (ERI), the following reliability indices were obtained: α = .759 for Efforts (Factor 1), and α = .780 for Rewards (Factor 2). The Composite Reliability Index (CRI) of the two factors measured by this version of the ERI were: CRI = .956 for F1 (Efforts), and CRI = .974 for F2 (Rewards).

#### Reliability and consistency of JCQ-20

As for the Job Content Questionnaire (JCQ), we found: α = .633 for Skill Discretion (Factor 1); α = .725 for Decision Authority (Factor 2); α = .761 for Psychological Job Demands (Factor 3); α = .886 for Supervisor Support (Factor 4); and α = .828 for Co-worker/Peer Support (Factor 5). The Composite Reliability Index (CRI) of the five factors assessed by this version of the JCQ were: CRI = .809 for F1 (Skill Discretion), CRI = .809 for F1 (Skill Discretion), CRI = .934 for F2 (Decision Authority), CRI = .965 for F3 (Psychological Job Demands), CRI = .983 for F4 (Supervisor Support), and CRI = .966 for F5 (Co-worker/Peer Support), showing suitable reliabilities for all the constructs.

### Factor correlations and convergent validity

In order to test the convergent validity of the ERI-10 and JCQ-20, all factors of both scales were contrasted with criterion variables (CVs), in order to assess their relationships (in terms of significance and association) in the glance of the existing evidences. Criterion variables were divided in three sets: the five factors of the validated version of COPSOQ-III (CV^a-e^); two health-related indicators: the psychological distress score of the GHQ-12 (CV^f^) and the self-rated health status item of COPSOQ-III (CV^g^); the number of occupational driving accidents suffered during the last two years (CV^h^). Based on the theoretical considerations and previous empirical evidence retrieved during the literature review (see *Background*), the main hypotheses for the correlations between ERI/JCQ factors and the criterion variables were:

(*i*) *ERI* vs. *JCQ:* ERI’s Efforts subscale will positively correlate to JCC’s Demands, and ERI’s Rewards will positively correlate to JCQ’s Control subscale. Also, the job stress indexes of both models will have a positive and significant correlation.

(*ii*) *ERI/JCQ and CVs*: The job stress indexes of both models (Effort-Reward Imbalance and Job Strain) will have correlations similar to the criterion variables, being significant and positive for CV^a^, CV^d^, CV^e^, CV^f^ and CV^h^, and significant and negative for CV^b^, CV^c^ and CV^g^). Almost all the Bivariate correlations between pairs of factors were, as hypothesized, statistically significant at level *p* < .001, directionally coherent and considerably large. Table 3 presents in detail the Pearson’s correlational coefficients (σ’s) among the full set of variables.

#### Convergent validity of ERI-10

The correlation between Efforts and Rewards (σ_*s*_ = −.444**) was negative and significant, and the correlation between Efforts and E-R Imbalance (ERI) was positive (σ_*s*_ = .826**). Also, ERI and Rewards had a significant and negative correlation (σ_*s*_ = −.851**), as hypothesized.

For what concerns the Job Demand-Control model (Karasek’s JCQ factors), all the relationships between the main factors of both scales were coherent and significant. Scores on Efforts and ERI were, respectively, positively correlated to Psychological Demands (σ_*s*_ = .624** and σ_*s*_ = .649**) and Job Strain (σ_*s*_ = .466** and σ_*s*_ = .619**), and negatively correlated to Control (σ_*s*_ = −.099^*^ and σ_*s*_ = −.277**) and Social Support (σ_*s*_ = −.282** and. σ_*s*_ = −.539**). On the other hand, Rewards’ scores were positively associated with Control (σ_*s*_ = .402**) and Social Support (σ_*s*_ = .629**), and negatively with Psychological Demands (σ_*s*_ = −.468**) and the Job Strain indicator (σ_*s*_ = −.565**).

As for the criterion variables, both Efforts and ERI (Imbalance) were found positively correlated to COPSOQ’s Demands (σ_*s*_ = .628** and σ_*s*_ = .695**), Job Insecurity (σ_*s*_ = .257** and σ_*s*_ = .342**) and Strain (σ_*s*_ = .475** and σ_*s*_ = .679**), and negatively to Influence and Development (σ_*s*_ = −.115** and σ_*s*_ = −.329**) and Leadership (σ_*s*_ = −.432** and σ_*s*_ = −.651**), while Rewards were negatively associated with Demands (σ_*s*_ = −.548**), Job Insecurity (σ_*s*_ = −.312**) and Strain (σ_*s*_ = −.667**), and positively correlated to Influence and Development (σ_*s*_ = .431**) and Leadership (σ_*s*_ = .667**). Moreover, occupational traffic crashes were positively correlated to the ERI ratio (σ_*s*_ = .084*) and negatively to Rewards (σ_*s*_ = −.093*), being all the directions of significant correlations coherent with theoretical assumptions.

#### Convergent validity of JCQ-20

The three main indicators of the Job Demand-Control (JDC) model presented significant and coherent correlations among them: Psychological Demands were negatively associated to Control (σ_*s*_ = −.141**) and positively to Job Strain (JS; σ_*s*_ = .791**), while Control showed a negative correlation to JS (σ_*s*_ = −.673**).

As for the set of criterion variables used, Psychological Demands shown a positive relationship to COPSOQ’s Demands (σ_*s*_ = .611**) and Strain (σ_*s*_ = .517**) subscales. On the other hand, Control had a positive correlation with both Influence and Development (σ_*s*_ = .513**) and Leadership (σ_*s*_ = .422**). The Karasek’s Job Strain (JS) index positively correlated to the COPSOQ’s Strain subscale (σ_*s*_ = .609**), the Psychological Distress indicator of the GHQ (σ_*s*_ = .365**), and negatively to the self-reported Health Status (σ_*s*_ = −.208**). Finally, occupational traffic crashes were significantly and negatively associated to Control (σ_*s*_ = −.133**), and positively with both Psychological Demands (σ_*s*_ = .088*) and the Job Strain index (σ_*s*_ = .170**), as theoretically expected. The full set of bivariate correlations is available at Table 6.

**Table 6:** Bivariate correlations (Spearman’s rho) between study factors and criterion variables

		ERI^F1^	ERI^F2^	ERI^F3^	JCQ^F1^	JCQ^F2^	JCQ^F3^	JCQ^F4^	JCQ^F5^	JCQ^F6^	JCQ^F7^	JCQ^F8^	CV^a^	CV^b^	CV^c^	CV^d^	CV^e^	CV^f^	CV^h^
*ERI factors*																			
ERI^F1^	Efforts	–																	
ERI^F2^	Rewards	−.444**	–																
ERI^F3^	ERI	.826**	−.851**	–															
*JCQ factors*																			
JCQ^F1^	Skill Discretion (F1)	−.012	.322**	−.194**	–														
JCQ^F2^	Decision Authority (F2)	−.099*	.378**	−.286**	.491**	–													
JCQ^F3^	Psychological Demands (F3)	.624**	−.468**	.649**	−.083*	−.176**	–												
JCQ^F4^	Supervisor Support (F4)	−.290**	.650**	−.557**	.356**	.445**	−.372**	–											
JCQ^F5^	Co-worker Support (F5)	−.211**	.427**	−.380**	.276**	.281**	−.250**	.537**	–										
JCQ^F6^	Control (F6)	−.099*	.402**	−.277**	.784**	.910**	−.141**	.469**	.312**	–									
JCQ^F7^	Social Support (F7)	−.282**	.629**	−.539**	.357**	.422**	−.359**	.905**	.824**	.452**	–								
JCQ^F8^	Job Strain (F8)	.466**	−.565**	.619**	−.489**	−.654**	.791**	−.531**	−.362**	−.673**	−.515**	–							
*Criterion variables*																			
CV^a^	Demands (F1)	.628**	−.548**	.695**	−.140**	−.188**	.611**	−.408**	−.381**	−.192**	−.445**	.532**	–						
CV^b^	Influence and Development (F2)	−.115**	.431**	−.329**	.428**	.457**	−.203**	.499**	.337**	.513**	.490**	−.447**	−.151**	–					
CV^c^	Relationships and Leadership (F3)	−.432**	.667**	−.651**	.312**	.404**	−.473**	.672**	.527**	.422**	.692**	−.576**	−.594**	.635**	–				
CV^d^	Job Insecurity (F4)	.257**	−.312**	.342**	−.043	−.029	.236**	−.160**	−.095*	−.042	−.154**	.168**	.410**	−.092*	−.301**	–			
CV^e^	Strain (F5)	.475**	−.667**	.679**	−.329**	−.400**	.517**	−.585**	−.462**	−.423**	−.602**	.609**	.713**	−.644**	−.849**	.392**	–		
CV^f^	Psychological Distress	.247**	−.279**	.321**	−.180**	−.250**	.308**	−.191**	−.200**	−.244**	−.217**	.365**	.406**	−.358**	−.399**	.249**	.547**	–	
CV^g^	Self-reported Health Status	−.149**	.260**	−.250**	.148**	.200**	−.138**	.232**	.225**	.189**	.261**	−.208**	−.318**	.273**	.382**	−.157**	−.479**	−.477**	–
CV^h^	Traffic Crashes (2 years)	.051	−.093*	.084*	−.116**	−.113**	.088*	−.094*	−.088*	−.133**	−.110**	.170**	.092*	−.094*	−.095*	−.048	.110**	.115**	−.099*

*Notes:*^a-e,g^COPSOQ factors; ^f^GHQ-12 indicator; ^h^Self-reported crash rate; *Correlation is significant at 0.05 level (2-tailed); **Correlation is significant at 0.01 level (2-tailed)

## Discussion

This study pursued the main aim of assessing the psychometric properties, convergent validity and internal consistency of two measures of occupational stress research (ERI and JCQ) among professional drivers. Overall, the outcomes of this empirical research confirm that both self-report tools keep a fairly adjusted factor configuration, adequate psychometric properties and convergent validity in regard to similar measures and complementary factors, such as health indicators and road safety outcomes, thus guaranteeing the methodological value of the validated versions of these questionnaires for their application among active workers of the transportation industry.

Precisely, the introduction of this article remarked the scarcity of validated (and reliable) self-report tools to address job stress from the perspective of psychosocial factors at work in highly vulnerable populations such as professional drivers. This gives a higher methodological value to the ones presented in this paper, if their short length, easy application and fair psychometric properties are considered. In regard to the validity of the ERI-10 and JCQ-20 factors, it is worth highlighting the high theoretical consistency of the original structure of both instruments with their validated version, highlighting the following particularities:

### Effort-reward imbalance inventory (ERI)

In the case of the ERI, all the lambda (λ) values, indicating the factor loading of each item, were higher than .30 (defined as a cut-off point). Also, the tested two-factorial model has shown a commendable fit, considering the model fit indices (i.e., CMIN/DF = 4.711; RMSEA = .072 - CI [.059–.085]; CFI = .957; and NFI = .947) [51].

Regarding the convergent validity of ERI factors (for guidance please see Table 6), this is an issue that has to be analyzed in the glance of other previous studies dealing with job stress in different populations, but especially with professional drivers, that document the relationship between the two root factors (*Efforts* and *Rewards*) and the *Effort-Reward Imbalance* ratio provided by the scale. In the first place, it is worth discussing some key findings in the *Efforts* subscale (F1). Scores in *Efforts* (measured by different versions of the ERI questionnaire) have been associated with health indicators of workers in different previous studies, also finding a negative relationship between job efforts and both mental and physical health issues [45, 62, 63].

Besides, *Efforts* were negatively associated to Rewards and positively to the ERI ratio, as observed in other studies [45, 63]. Further, a recent systematic review on the Effort-Reward Imbalance among health workers, performed by Nguyen Van et al. [64], found that, in more than 40 different empirical studies, *Rewards* (F2) are often perceived as lower than the efforts put in the development of their job tasks, suggesting the need of empirically-based interventions on workplace environmental conditions and job stress. Finally, the convergent validity of the three factors analyzed in the instrument was satisfactorily tested through the assessment of significant and coherent correlations with criterion variables, in accordance with the previous empirical sources of evidence, in terms of: *a*) job stress measured through other similar inventories, such as the JCQ and COPSOQ [47, 65], *b*) physical and mental health issues [13, 42, 47], and *c*) occupational accidents [39, 66].

### Job content questionnaire (JCQ)

As for the JCQ, the baseline model has displayed a relatively good (but improvable) structure that showed substantial improvements once two items from the *Skill Discretion* subscale, that presented lambda values <.03 (low factor loadings), were deleted. This led to a retained model with highly suitable indices (CMIN/DF = 3.120; RMSEA = .054 - CI [.049–.060]; CFI = .943; and NFI = .918), suggesting an optimal fit to the data.

As for the convergent validity of the JCQ, the associations found between JDC model’s main subscales and variables measured through similar and/or complementary instruments show coherence with the theoretical assumptions followed by our study, and further empirical findings provided by previous studies in the field. First of all, scores in Psychological Demands were inversely associated with Control, and positively with both COPSOQ’s Demands subscale and the Job Strain (JS) index, that is the stress indicator of the model [3, 11]. JS is also positively correlated to the scores obtained in the Strain subscale of COPSOQ, a directly convergent measure.

Moreover, higher scores in the JS index have shown to be associated to poorer outcomes in terms of mental health assessed through questionnaire-based methods, such as different versions of the GHQ [67–69] and other self-reported measures aimed at assessing the health status of workers [70–72], keeping the same directional associations than the found in this study. Also, previous research based in cohort studies have also documented significant association between (and even a predictive value of) Job Strain and, e.g., coronary heart disease [2], type-II diabetes [73, 74] and musculoskeletal symptoms [15, 75], that makes sense if the fact that work environment of professional drivers is also characterized by continuous physical and ergonomic demands is considered [18, 39, 46, 76]. Lastly, the number of occupational accidents suffered “at the wheel” by professional drivers has shown a significant association to Job Strain. This is, perhaps, one of the most frequently empirical finding in studies using approaches similar to Karasek’s JDC in professional drivers, in terms of driving performance and occupational safety [3, 19, 39].

It is also important to point in the fact that, given the structural modifications performed on the JCQ (originally containing other two items that were dismissed), the scoring methodology for calculating the Control (F6) and the Job Strain index (JS; F8) has been successfully adapted to the item ratio between F1 (Skill Discretion) and F2 (Decision Authority), and between F6 (Control) and F3 (Demands), necessarily being 1:1 for the calculation of the Job Strain index.

#### Limitations of the study and further research

Although this research used a considerably large (although not representative) study sample, the statistical parameters and model fit coefficients were adequately verified, and the quality and value of the questionnaires had been previously supported by many empirical studies, some both methodological and qualitative biasing sources should be considered. Firstly, the research was carried out by means of self-report-based data, and several studies have shown how self-report measures may carry different biases, such as acquiescent answers (i.e., the total agreement of participants with the presented questions), social desirability and lack of sincerity, especially considering that most of the questionnaires were applied at the workplace, in the companies where the drivers were working. Furthermore, positive/negative affects/mood may impact the response style of participants, especially when addressing issues that may seem sensitive, such as health issues [77] and occupational traffic crashes, even when responding to anonymous questionnaires, as pointed out by Chai et al. [78] and Af Wåhlberg [79] in previous studies dealing with drivers and their road safety outcomes.

Regarding the questionnaire contents, it is worth remarking that, although standardized scales such as the ERI and JCQ (in their different versions) have a demonstrated to be valuable for measuring psychosocial job-related factors in different occupational groups, they fail to address specific stressors and hazardous working conditions that are particular to each profession. Thus, it is advisable to use these tools together with an assessment of the specific (e.g.) stressors, demands and reward modalities of the job, perhaps adding additional short scales and/or qualitative questions that may strengthen interpretations and outcome comparisons, as performed in recent research carried out with professional drivers [80]. Additionally, the authors would like to promise that they will consider all the issues that cannot be fixed a posteriori in this study for further ones.

Finally, this study used a transversal (or cross-sectional) design, which means that the outcomes are obtained from a single measurement moment. Although it methodologically allows for the fulfillment of the study aim, the use of multiple measures may contribute to test the stability, consistency and invariance of the instrument over time (e.g., test-retest reliability), strategy that (even though encompassing higher efforts and further measurements) may represent further insights on the study of psychosocial factors at work following the ERI and JCQ approaches.

## Conclusion

The findings of this study support the hypothesis that the validated versions of both the ERI and JCQ scales, used for assessing job stress from different theoretical approaches, present adequate structural, psychometric and practical features; this makes them suitable for being applied to the study of the phenomenon among professional drivers, as well as workers employed in other similar occupations and facing similar task-related factors. Such as the ones addressed by the ERI and JDC models (for instance, excessive efforts, psychological demands, time pressure, and lack of rewards and/or social support). Furthermore, the validated scales keep an adequate convergent validity with criterion variables extracted from similar measures (such as the COPSOQ-III and the GHQ) and occupational (road) safety indicators.

Also, it is important to remark, given both the extensive previous background that exists in this regard and the results of this study, that occupational stress research and intervention can be a useful step to strengthen the road safety outcomes of professional drivers, that nowadays constitute a public health concern. Thus, and keeping in mind the reduced length of ERI-10 and JCQ-20, and several other studies supporting their scientific value, these questionnaires can be useful for performing occupational research focused on psychosocial factors at work, and for designing evidence-based interventions aimed at improving the environmental conditions of the job, as well as the health and safety outcomes of workers in this hazardous industry.

### Application (practical implications)

This study provides the validated versions of two widely used self-report-based psychosocial research tools for assessing job stress, with an adapted structure, for their using among professional drivers: Siegrist’s ERI and Karasek’s JCQ.

The shortened versions of these instruments, that present fair psychometric properties and convergent validity, can be useful for occupational researchers and practitioners studying stress-related issues from the perspective of human factors at occupational and public health settings.

## Data Availability

The data that support the findings of this study are available from the corresponding author (S.A.U.), upon reasonable request.

## Appendix 1 Exploratory Factor Analysis: Item factor weights for ERI and JCQ

**Table 7:** Exploratory Factor Analysis (EFA): Item factor loadings for the Effort-Reward Imbalance (ERI) inventory – rotated matrix (Promax)

ERI Item	Efforts	Rewards
**ERI2**	.802	−.213
**ERI1**	.745	−.273
**ERI3**	.706	−.136
**ERI10**	−.303	.824
**ERI8**	−.389	.815
**ERI4**	−.287	.778
**ERI9**	−.242	.757
**ERI6**	−.485	.695
**ERI7**	−.362	.592
**ERI5**	−.299	.463

**Table 8:** Exploratory Factor Analysis (EFA): Item factor loadings for the Job Content Questionnaire (JCQ) – rotated matrix (Promax)

JCQ Item	Supervisor Support	Co-worker Support	Psychological Job Demands	Decision Authority	Skill Discretion
**JCQ14**	.883	.437	−.273	.419	.152
**JCQ15**	.857	.416	−.261	.393	.170
**JCQ16**	.854	.507	−.329	.376	.208
**JCQ13**	.846	.482	−.317	.388	.209
**JCQ19**	.450	.844	−.174	.218	.140
**JCQ20**	.391	.828	−.175	.189	−.016
**JCQ18**	.510	.827	−.274	.271	.223
**JCQ17**	.364	.743	−.196	.244	.224
**JCQ9**	−.144	−.132	.829	−.026	.017
**JCQ8**	−.385	−.332	.806	−.270	.057
**JCQ10**	−.197	−.078	.805	−.090	.132
**JCQ12**	−.316	−.277	.593	−.301	.199
**JCQ11**	.212	.163	−.295	.016	.170
**JCQ3**	.313	.300	−.231	.787	.122
**JCQ6**	.555	.291	−.209	.784	.314
**JCQ4**	.210	.067	.018	.741	.148
**JCQ2**	.169	.089	.104	.224	.789
**JCQ1**	.311	.236	−.163	.456	.739
**JCQ5**	.142	.115	.063	.106	.716
**JCQ7**	.208	.213	−.080	.424	.562

## Abbreviations

JCQ: Job Content Questionnaire
JDC: Job Demand-Control (model)
JS: Job Strain (job stress indicator of the JDC model)
ERI: Effort-Reward Imbalance (model and questionnaire)
COPSOQ: Copenhagen Psychosocial Questionnaire
GHQ: General Health Questionnaire
